# A Rare Presentation of Metastatic Prostate Cancer With Recurrent Malignant Ascites: A Case Report and Literature Review

**DOI:** 10.7759/cureus.96993

**Published:** 2025-11-16

**Authors:** Yanming Wu, Sunydip Gill, Leen Khoury, Mahtab Zangui, Mary Hanna, Edwin Chiu

**Affiliations:** 1 Internal Medicine, State University of New York Downstate Health Sciences University, Brooklyn, USA; 2 Hematology and Medical Oncology, State University of New York Downstate Health Sciences University, Brooklyn, USA; 3 Pathology, State University of New York Downstate Health Sciences University, Brooklyn, USA

**Keywords:** androgen-deprivation therapy, malignant ascites, peritoneal carcinomatosis, prostate cancer, prostate specific antigen

## Abstract

Peritoneal carcinomatosis with malignant ascites is a rare and aggressive presentation of advanced prostate cancer, often linked to a poor prognosis. Here, a case of a 58-year-old African American male presenting with ascites as the initial manifestation of prostate cancer is reported. Imaging revealed peritoneal carcinomatosis with concomitant pelvic lymph nodes and bone metastases. Cytologic analysis of the ascitic fluid confirmed prostate origin. The patient underwent androgen deprivation therapy and systemic chemotherapy with docetaxel followed by cabazitaxel. Despite treatment, the disease progressed rapidly with rising prostate-specific antigen levels and recurrent ascites, and he ultimately passed away 15 months after diagnosis. A literature review of similar cases is included, highlighting the poor clinical outcome posed by this rare presentation. Currently, there are no standardized guidelines for managing malignant ascites in prostate cancer. This case underscores the importance of multidisciplinary care addressing both oncologic and social factors. Further research is warranted to elucidate the mechanisms of peritoneal dissemination, improve early detection, and develop tailored therapeutic strategies for prostate cancer presenting with malignant ascites.

## Introduction

Prostate cancer is the most common non-cutaneous malignancy in men and the second leading cause of cancer-related mortality in the United States [1]. The overall prognosis is generally good, particularly when detected early, with a 10-year relative survival rate approaching 100%. However, approximately 6% of patients present with metastatic disease at diagnosis, for whom the five-year survival rate drops to 29%. The most frequent sites of metastasis include the bones, lymph nodes, liver, and thorax [2]. By comparison, peritoneal carcinomatosis is a rare manifestation of advanced prostate cancer, with most reports in the literature consisting of individual case studies.

Ascites is typically seen as a sequela of portal hypertension and cirrhosis, but malignancy-related ascites accounts for approximately 10% of cases [3]. The most common malignancies associated with peritoneal carcinomatosis include ovarian, colorectal, gastric, pancreatic, breast, and lung cancers [4]. Peritoneal involvement in prostate cancer is extremely rare and is often associated with the development of castration-resistant disease or high-risk prostate cancer, which portends a poor clinical outcome. Given the rarity of this presentation, the mechanisms underlying peritoneal dissemination in prostate cancer remain largely unexplored. Proposed pathways include iatrogenic seeding after surgery, hematological or lymphatic spread, direct invasion, and transcoelomic seeding driven by mechanical and molecular factors [5-7].

Here, a rare case of recurrent malignant ascites as the initial presentation of prostate cancer is reported. This case adds to the growing body of literature on peritoneal carcinomatosis in prostate cancer and highlights the diagnostic and management challenges associated with this rare presentation.

## Case presentation

A 58-year-old African American male with a history of alcohol use disorder presented with failure to thrive, a 40-lb weight loss over one year, generalized abdominal pain, and two days of coffee-ground emesis. On examination, he was hemodynamically stable with a distended and diffusely tender abdomen. Laboratory workup was largely unremarkable except for a markedly elevated prostate-specific antigen (PSA) of 567.8 ng/mL (reference range: 0.0-4.0 ng/mL).

**Figure 1:**
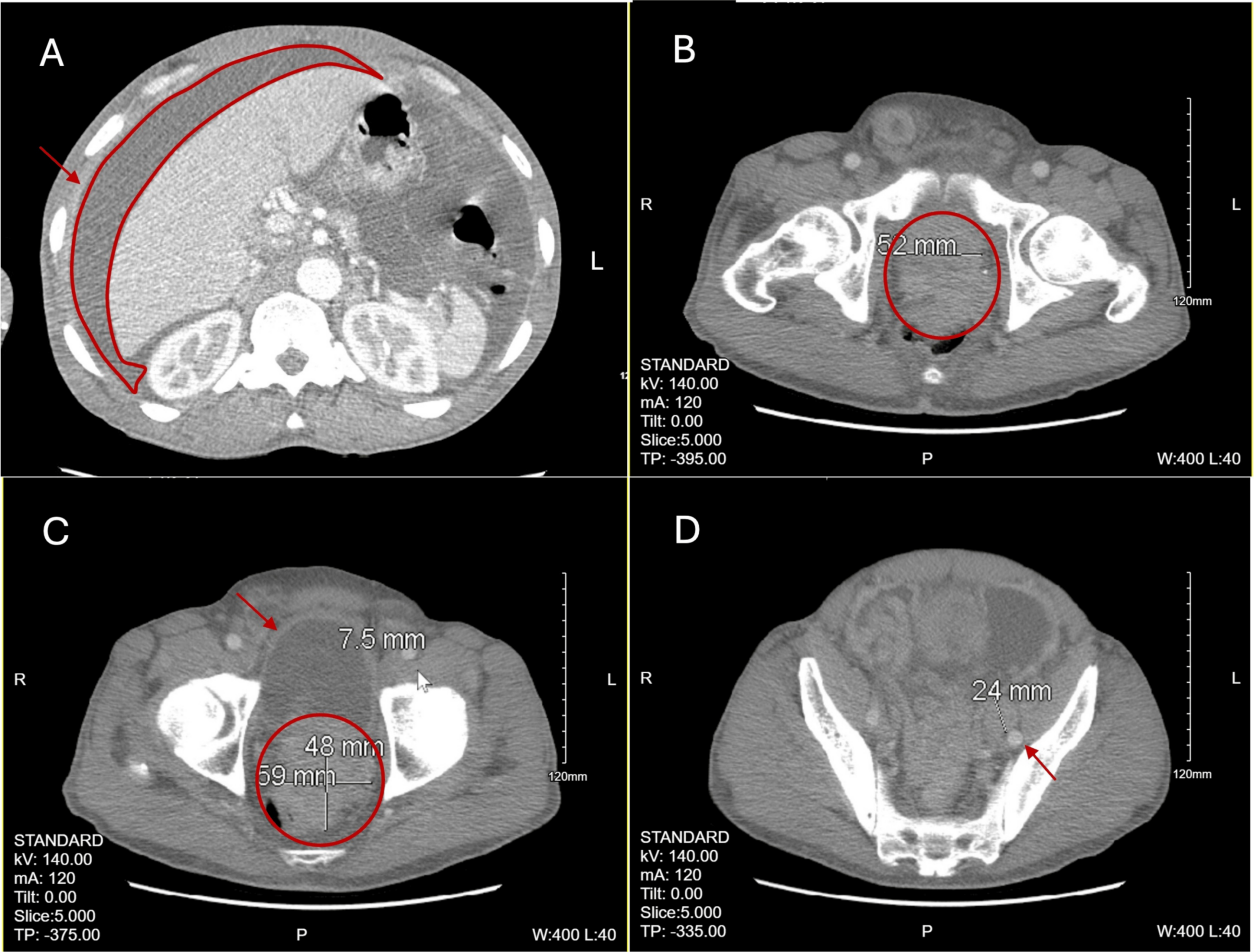
Patient’s CT abdomen and pelvis with IV contrast. (A) Massive ascites (circle) with enhancement of the peritoneum (arrow), (B) enlarged prostate gland (circle), (C) continuous soft tissue mass extending from the prostate gland, involving the rectum (circle), thickened bladder wall (arrow), (D) significant pelvic lymphadenopathy (arrow).

A contrast-enhanced CT of the abdomen and pelvis revealed a 5.2 cm prostate gland with a 5.1 cm x 4.8 cm enhancing soft tissue mass extending posteriorly and superiorly, invading the rectum and sigmoid colon. There were significant pelvic lymphadenopathy and ascites with peritoneum enhancement (Figure 1). A nuclear medicine (NM) bone scan demonstrated multifocal osseous metastases. Prostate biopsy confirmed prostate adenocarcinoma, stage IVB (cT4, cN1, cM1b), with a Gleason Score of 4+5=9. The patient was started on bicalutamide 50mg daily (adherent for approximately three months) and leuprolide 45mg injection planned for every six months. Two months after treatment initiation, his PSA level decreased to 7.93 ng/mL. Multiple paracenteses were preformed but were noncontributory. Addition of abiraterone/prednisone or docetaxel was deferred due to the patient’s heart failure reduced ejection fraction (HFrEF) with EF at 35%.

**Figure 2:**
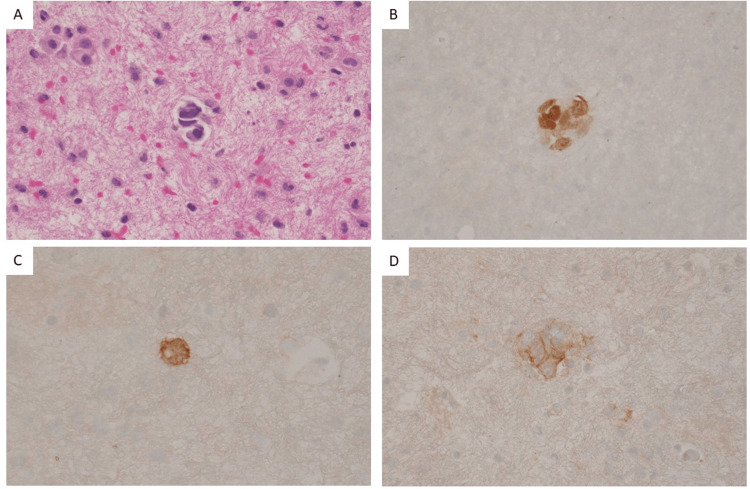
Cytology of ascitic fluid demonstrating peritoneal carcinomatosis from prostate cancer. Cytology of ascitic fluid demonstrating peritoneal carcinomatosis from prostate cancer. (A) Clusters of pleomorphic neoplastic cells with irregular nuclear membrane and prominent nucleoli on hematoxylin and eosin (H&E) stain. Immunohistochemistry shows tumor cells positive for (B) NKX3.1 (prostate-specific marker), (C) MOC-31 (adenocarcinoma marker), and (D) Ber-EP4 (adenocarcinoma marker) (Original magnification, 400x).

Four months later, after being lost to follow-up due to residential instability and financial hardship, the patient re-presented with recurrent ascites. He had not been compliant with bicalutamide or leuprolide. His PSA had risen to 90.28 ng/mL. Diagnostic and therapeutic paracentesis yielded 2.8L of serosanguinous fluid with 3000 RBCs/μL (normally absent), albumin 2.0 g/dL (variable, comparison with serum level is recommended), total protein 4.2 g/dL (transudate <2.5 g/dL, exudate ≥2.5g/dL), glucose 87 mg/dL (normally similar to serum level), and negative gram stain. The serum albumin was 2.9 g/dL (reference range: 3.3-6.1 g/dL), resulting in a serum-ascites albumin gradient (SAAG) < 1.1, consistent with an exudative process. Cytology was positive for adenocarcinoma (Ber-EP4 and MOC-31 positive), with NKX3.1 immunoreactivity confirming peritoneal carcinomatosis of prostate origin (Figure 2). MRI ruled out epidural involvement. Intensification of androgen deprivation therapy (ADT) including abiraterone and prednisone was planned but interrupted again due to loss of follow-up.

Two months later, the patient returned with worsening ascites requiring frequent paracenteses. His PSA had increased to 633 ng/mL. Owing to disease progression, he was restarted on bicalutamide followed by leuprolide. After obtaining cardiology clearance (EF 30%, moderate mitral regurgitation), he began chemotherapy with docetaxel (50% dose reduction for the first two cycles and full dose for the next three) combined with prednisone 5mg twice daily per CHAARTED and STAMPEDE trials’ evidence [8,9]. He completed five of the six planned cycles, with the last cycle withheld due to neutropenia. Because of PSA progression and a new liver lesion noted on CT, treatment was transitioned to cabazitaxel at 20 mg/m^2^ in combination with prednisone 10mg once daily per TROPIC and PROSELICA trials’ evidence [10,11]. After three cycles, the patient developed septic shock and worsening kidney function and died two weeks after his last treatment.

## Discussion

Prostate cancer is the most commonly diagnosed malignancy among men in the United States and generally carries a favorable prognosis [1]. However, peritoneal involvement is extremely rare and typically indicates rapid progression with a poor outcome. In a retrospective registry study, only 1.9% of patients with prostate cancer who died had undergone paracentesis for ascites during the last year of life, with ascites most often occurring in the terminal phase rather than at initial diagnosis [12].

With advancements of systemic therapies, including ADT combined with androgen receptor pathway inhibitors (ARPIs) and chemotherapy, the median overall survival (OS) for *de novo* metastatic prostate cancer has improved significantly over the past two decades, although it remains approximately 30 months [13]. We reviewed 13 additional published case reports describing prostate cancer with malignant ascites (Table 1). Among these cases, 46% of patients presented with ascites at diagnosis, and more than half experienced disease progression despite treatment, underscoring both the rarity of this manifestation and its poor prognosis.

**Table 1:** Review of the case reports of prostate cancer with malignant ascites ADT: Androgen deprivation therapy; TURP: Transurethral resection.

No	Authors	Age	Site of Metastasis (apart from peritoneal / omentum)	Stage at initial diagnosis	Treatment		Time from Diagnosis to Ascites	Ascites Response	Outcome
					Before Ascites	After Ascites (apart from paracentesis)			
1	Ifeanyl Ani, et al. [18]	57	Bone	Stage IV	N/A	ADT (bicalutamide + leuprolide)	0	Symptomatic from ascites	Multiple admissions due to ascites
2	Virginia Visconti, et al. [19]	77	Bone	Stage IV	N/A	ADT (bicalutamide + leuprolide)	0	Improvement within 3 months	Alive at 3 months
3	Dimitrios Petrakis, et al. [20]	76	Lymph nodes	Not mentioned	TURP + ADT (bicalutamide + leuprolide)	Docetaxel + prednisone	16 years	Asymptomatic at 10 months	Alive at 10 months
4	Aparajit Ram Venkateswaran, et al. [21]	78	Lung, bone, bladder, lymph nodes	Stage II	Radiotherapy, ADT (bicalutamide + leuprolide)	Docetaxel	12 years	Progression	Deceased 3 months after ascites and obstructive shock
5	Serene A. Tareen, et al. [22]	70	Bladder, seminal vesicle, liver	Stage IV	N/A	ADT (bicalutamide + leuprolide), docetaxel, abiraterone + prednisone, cabazitaxel	0	Progression	Transitioned to hospice after 6 months
6	James Y Tsai, et al. [23]	68	Rectum	Stage IV	Goserelin + flutamide, interferon alpha-2b + toremifene	Not mentioned	1 year 8 months	Progression	Deceased 4 months after ascites
7	Hee Ryeong Jang, et al. [24]	61	Bladder, ureter	Stage IV	ADT (Leuprolide + flutamide)	Abiraterone	27 months	Improvement	Alive at 1 month
8	Rajeev Saini, et al. [25]	65	N/A	Not mentioned	Bilateral orchiectomy, bicalutamide, fosfestrol, ketoconazole + prednisolone	Taxotere-based chemotherapy	10 years	Not mentioned	Not mentioned
9	Samuel P Benedict, et al. [26]	67	Bladder	Not mentioned	Bilateral orchiectomy + TURP + bicalutamide	Docetaxel	22 months	Improvement	Alive at docetaxel cycle 2
10	Shabnam Samankan, et al. [27]	84	N/A	stage IV	N/A	Docetaxel, enzalutamide	0	Improvement	Alive after 10 doses of docetaxel
11	Stamatis S Papadatos, et al. [28]	69	Bone	stage IV	N/A	Radiation + bicalutamide + triptorelin	0	Progression	Deceased 3 months after from acute pulmonary edema
12	Mawuenyo Attawa Oyortey, et al. [29]	64	Perineural invasion, bone	stage IV	N/A	Bilateral total orchidectomy, docetaxel	0	Became castration-resistant	Alive after orchidectomy
13	Fahd Khan, et al. [30]	66	Kidney, liver	stage III	Neoadjuvant antiandrogen therapy + radiotherapy + antiandrogen therapy	palliative care	7 years	Progression	Deceased 6 weeks after ascites

Currently, there are no prostate cancer-specific guidelines for managing malignant ascites. Management is generally extrapolated from broader oncology and palliative care practices, with a primary focus on symptom relief and quality of life. Diagnostic challenges further complicate care; the false-negative rate of ascitic fluid cytology in malignancy can be as high as 42% [14], as seen in our case, potentially contributing to the delayed diagnosis when ascites is the initial manifestation.

Systemic treatment options for prostate cancer with malignant ascites include ADT, ARPIs, chemotherapy, and radiotherapy, tailored to disease burden, prior treatments, progression pattern, and patient performance status [15]. Symptomatic ascites is typically managed with serial paracenteses, careful fluid balance, and diuretics as clinically indicated. In select cases, more aggressive or investigational interventions, such as intraperitoneal chemotherapy [16] or PSMA-targeted radioligand therapy (e.g., [¹⁷⁷Lu]Lu-PSMA-617, as evaluated in the VISION trial) [17], have been reported, though these approaches remain outside standard practice.

Given the limited evidence and lack of consensus guidelines, a multidisciplinary and individualized approach is essential. Management should align with the patient’s goals of care, functional status, and symptom burden, with an emphasis on optimizing quality of life in advanced disease.

## Conclusions

We present a rare case of prostate cancer with malignant ascites as an initial manifestation. The patient responded initially to leuprolide and bicalutamide, with a rapid PSA decline within three months. However, disease progression occurred following a prolonged lapse in follow-up, manifested by recurrent ascites and rising PSA levels. The patient was later treated with ARPI and chemotherapy with docetaxel followed by cabazitaxel. He ultimately passed away 15 months after the initial presentation, a survival significantly shorter than reported in population-based studies.

Peritoneal carcinomatosis from prostate cancer represents a rare and aggressive disease course, often associated with a high-grade or castration-resistant tumor. Malignant ascites and peritoneal carcinomatosis from prostate cancer correlate with markedly higher mortality and shorter survival, often measured in weeks to months. Clinicians should maintain a high index of suspicion for metastatic prostate cancer when encountering unexplained ascites. In this case, treatment interruptions totaling six months, driven by loss of follow-up, nonadherence to treatment as planned, socioeconomic barriers such as unstable housing and limited family support, worsening cardiac function, and complications of progressive cancer, all of which further contributed to the poor outcome. This underscores the importance of multidisciplinary care that addresses not only oncologic management but also comorbidities, psychosocial support, and palliative needs.

Further research is needed to better understand the biological mechanisms of peritoneal dissemination in prostate cancer. Studies exploring molecular pathways governing cell adhesion, epithelial-mesenchymal transition, and tumor-peritoneal microenvironment interactions may clarify this atypical metastatic pattern. Integrating genomic and transcriptomic profiling into such rare cases could help identify predictive markers and potential therapeutic targets, guiding precision management for prostate cancer with malignant ascites.
